# Evaluation of oral manifestations and mandibular bone fractal dimension in pediatric inflammatory bowel disease

**DOI:** 10.3389/fped.2026.1882737

**Published:** 2026-08-03

**Authors:** Muhammed Abdullah Çege, Ecem Elif Çege, Yakup Doğru, Neslihan Ekşi, Burak Tula, Taner Özgür, Ahmet Zeki Berk, Fatih Eroğlu, Nergiz Sevinç

**Affiliations:** 1 Department of Oral and Maxillofacial Surgery, Faculty of Dentistry, Karabuk University, Karabuk, Türkiye; 2 Department of Pediatric Dentistry, Faculty of Dentistry, Karabuk University, Karabuk, Türkiye; 3 Department of Pediatric Dentistry, Faculty of Dentistry, Bursa Uludag University, Bursa, Türkiye; 4 Department of Pediatric Gastroenterology, Faculty of Medicine, Karabuk University, Karabuk, Türkiye; 5 Department of Pediatrics and Pediatric Gastroenterology, Faculty of Medicine, Bursa Uludag University, Bursa, Türkiye; 6 Department of Radiology, Karabuk Training and Research Hospital, Karabuk, Türkiye; 7 Department of Public Health, Faculty of Medicine, Karabuk University, Karabuk, Türkiye

**Keywords:** Crohn's disease, fractal dimension analysis, oral manifestations, oral mucosal lesions, pediatric inflammatory bowel disease, ulcerative colitis

## Abstract

**Objective:**

This study aimed to evaluate oral manifestations, caries experience, and mandibular trabecular bone microarchitecture using fractal dimension (FD) analysis in children with inflammatory bowel disease (IBD), and to compare these findings with a healthy control group.

**Methods:**

In this multicenter case-control study, children with inflammatory bowel disease (IBD) and age- and gender-matched healthy controls underwent oral mucosal examination, dmft/DMFT indices, and the Community Periodontal Index of Treatment Needs (CPITN). Mandibular trabecular bone microarchitecture was evaluated using fractal dimension (FD) analysis applied to digital panoramic radiographs. The influence of medication type and duration on oral findings was additionally investigated.

**Results:**

The study included 34 children with IBD (10 had Crohn's disease and 24 had ulcerative colitis) and 34 age- and gender-matched healthy controls. No significant differences were observed between the groups regarding caries experience or CPITN scores (*p* > 0.05). FD values were significantly lower in the IBD group at the condyle (*p* = 0.047) and molar (*p* < 0.001) regions. Children with Crohn's disease demonstrated lower condylar FD values compared to patients with ulcerative colitis (*p* = 0.046). Intraoral lesions were more frequent in Crohn's disease, with aphthous ulcers being the most common finding. No significant association was found between medication duration and oral findings (*p* > 0.05).

**Conclusion:**

Children with IBD, particularly those with Crohn's disease, exhibited reduced mandibular trabecular bone complexity and increased oral mucosal involvement. Fractal dimension analysis may represent a useful non-invasive tool for assessing mandibular bone microarchitecture in this population.

## 1 Introduction

Inflammatory bowel disease (IBD) is the most common chronic gastrointestinal disorder in children and primarily encompasses two main clinical subtypes: Crohn's disease (CD) and ulcerative colitis (UC). The epidemiology of pediatric IBD varies considerably across geographic regions. Annual incidence rates have been reported to range from 1.59 to 10.9 cases per 100,000 children in Europe and from 11.4 to 13.2 cases per 100,000 children in North America, whereas substantially lower rates have been observed in Asia and the Middle East (0.47–2.16 cases per 100,000 children) (1).

Pediatric-onset IBD is recognized as a systemic condition that affects not only the gastrointestinal tract but also growth and development, nutritional status, and various extraintestinal organ systems (2). The management of IBD involves several pharmacological approaches, including aminosalicylates, corticosteroids, immunomodulators, and biological agents; the long-term use of these therapies is of particular concern regarding systemic side effects, especially in children (3).

Oral findings in patients with IBD are among the extraintestinal manifestations of the disease and may precede gastrointestinal symptoms in some cases. The lips, buccal mucosa, and gingiva are the most common sites of oral manifestations in pediatric IBD. Aphthous ulcers, angular cheilitis, mucosal edema, erythema, gingivitis, and cobblestone appearance of the mucosa particularly observed in CD are among the most frequently reported oral findings (4, 5). These lesions may be associated with disease activity, nutritional deficiencies, saliva pooling at the corners of the mouth, and immunosuppressive therapies. Furthermore, various studies have reported a higher prevalence of dental caries and periodontal disease in patients with IBD (6, 7).

The systemic inflammatory nature of IBD may affect not only soft tissues but also bone metabolism and bone microarchitecture. Factors such as malnutrition, delayed puberty, reduced physical activity, and impaired nutrient absorption in children with IBD may lead to deterioration of trabecular bone structure. Active intestinal inflammation in particular has been reported to be one of the primary determinants of alterations in bone metabolism; proinflammatory cytokines of intestinal origin and systemic corticosteroids frequently used in IBD treatment have been shown to suppress osteoblast activity, thereby reducing bone formation and adversely affecting bone cell functions (8). As a result of these mechanisms, decreased bone mineral density and alterations in trabecular bone architecture may occur in children with IBD (9, 10).

Fractal dimension (FD) analysis is a method based on the mathematical modeling of complex and irregular structures. This method is used in medicine and dentistry to objectively evaluate structural changes in tissues and organs through quantitative data and to detect pathological changes at an early stage. One of its most important advantages is its ability to provide quantitative data non-invasively compared to conventional imaging techniques (11, 12). Considering that bone metabolism may be adversely affected in children diagnosed with IBD due to factors such as chronic systemic inflammation, malnutrition, impaired absorption of vitamins and minerals, delayed puberty, reduced physical activity, and medications used in the treatment of the disease, FD analysis is anticipated to be an appropriate and non-invasive method for evaluating microstructural changes that may occur in bone tissue. Although studies exist in the literature in which FD analysis has been used in patients with IBD to evaluate cellular and tissue-level changes, particularly in intestinal epithelium and extracellular matrix structure (13, 14), literature regarding the evaluation of mandibular trabecular bone structure using FD analysis in children with IBD remains extremely limited.

This study aimed to achieve several objectives. First, to evaluate oral findings, caries experience, and mandibular trabecular bone structure using FD analysis in children diagnosed with IBD. Second, to examine the effects of medication type and duration of medication use on oral findings, including dmft/DMFT indices, the Community Periodontal Index of Treatment Needs (CPITN), and intraoral lesions. Third, to compare these findings with those of a healthy control group. The null hypothesis of the study is that there is no statistically significant difference between children diagnosed with IBD and the healthy control group in terms of oral findings, caries experience (dmft/DMFT indices), and FD values of mandibular trabecular bone. This study has the distinction of being the first study in which mandibular trabecular bone has been evaluated using FD analysis in children with IBD.

## 2 Methods

### 2.1 Study design and ethical approval

This research is an observational study consisting of cross-sectional and retrospective components. Oral findings, caries experience, and mandibular trabecular bone structure were evaluated using FD analysis in children diagnosed with IBD. The study is a multicenter research conducted across Karabuk and Bursa provinces, in collaboration with Karabuk Oral and Dental Health Training and Research Hospital.

Ethical approval for this study was obtained from the Karabuk University Non-Interventional Clinical Research Ethics Committee (Decision No: 2026/2722; Date: 10 February 2026), and institutional permission for data collection was obtained from Karabuk Oral and Dental Health Training and Research Hospital (Permission No: E-21767321-501.07.01-319446068). Written and verbal informed consent was obtained from all participants or their legal guardians.

### 2.2 Sample size

Relevant studies that could be used for sample size estimation were reviewed, and the sample size yielding the largest number, based on the statistical methods to be applied in line with the main hypotheses, was taken into consideration. Although it has been reported that approximately 20 cases per group are sufficient in the current literature (15), the sample size in this study was set at 25 for both the patient and control groups in order to increase statistical reliability. The sample size was calculated using G*Power 3.1 software, based on a 95% confidence level (*α* = 0.05) and 80% statistical power (1 − *β* = 0.80).

### 2.3 Study population

The study group consisted of children who presented to the Oral and Dental Health Hospitals for routine clinical examinations and were determined to have been diagnosed with IBD, such as CD or UC, by the pediatric gastroenterology unit based on the anamnesis obtained. Additionally, patients with a digital panoramic radiograph taken within the last 6 months available in the hospital archive were included in the study, and no additional radiograph was taken from these patients for research purposes.

The control group consisted of healthy children without systemic or chronic disease who presented to both Oral and Dental Health Training and Research Hospitals, were compatible with the study group in terms of age and gender, and had digital panoramic radiographs available. The control group was formed by selecting the first healthy individual compatible in terms of age and gender who presented to the clinic immediately after each study group patient was enrolled. Selection bias was minimized through this consecutive matching method.

### 2.4 Inclusion criteria

- Aged between 7 and 19 years,
- For the study group, diagnosed with IBD (CD or UC) by pediatric gastroenterology,
- Availability of a digital panoramic radiograph taken within the last 6 months following diagnosis and archived in the hospital records,
- For the control group, individuals matched with the study group in terms of age and gender and with no history of any systemic disease were included in the study.

### 2.5 Exclusion criteria

- Children with systemic diseases in addition to IBD (i.e., co-existing comorbid conditions),
- Panoramic radiographs with insufficient image quality that would preclude fractal analysis, including those containing horizontal or vertical distortions, or metal and motion artifacts,
- Panoramic radiographs in which the mental foramen could not be clearly identified,
- Panoramic radiographs in which the region of interest (ROI) areas selected for fractal analysis could not be clearly visualized or in which superimposition of surrounding anatomical structures was present were excluded from the study.

### 2.6 Clinical data assessment

Demographic data such as age and gender, time of diagnosis, medications used since diagnosis, and intraoral findings of the disease were recorded by two experienced specialist pediatric dentists for patients presenting to the Oral and Dental Health Hospitals for routine clinical examination. Among oral mucosal findings, the presence of cobblestone appearance, aphthous ulcers, angular cheilitis, and gingivitis was evaluated. Caries assessment was performed through visual examination and review of previous radiographs. The dmft and DMFT indices were used to assess caries experience based on the World Health Organization (WHO) criteria. Accordingly, carious lesions, tooth losses due to caries, and the presence of restorations were recorded. The dmft index was calculated for primary teeth and the DMFT index for permanent teeth, while in patients in the mixed dentition period, dmft and DMFT values were evaluated together.

The CPITN index was used to assess periodontal health (16). After dividing the oral cavity into six sextants, the highest score in each sextant was recorded. Scoring was performed as follows: 0, healthy periodontium; 1, bleeding on probing; 2, presence of calculus; 3, periodontal pocket depth ≥4 mm and <6 mm; 4, periodontal pocket depth ≥6 mm. All examinations were performed by the same clinicians using a standard WHO periodontal probe.

### 2.7 Radiographic evaluation and fractal analysis

**Figure 1:**
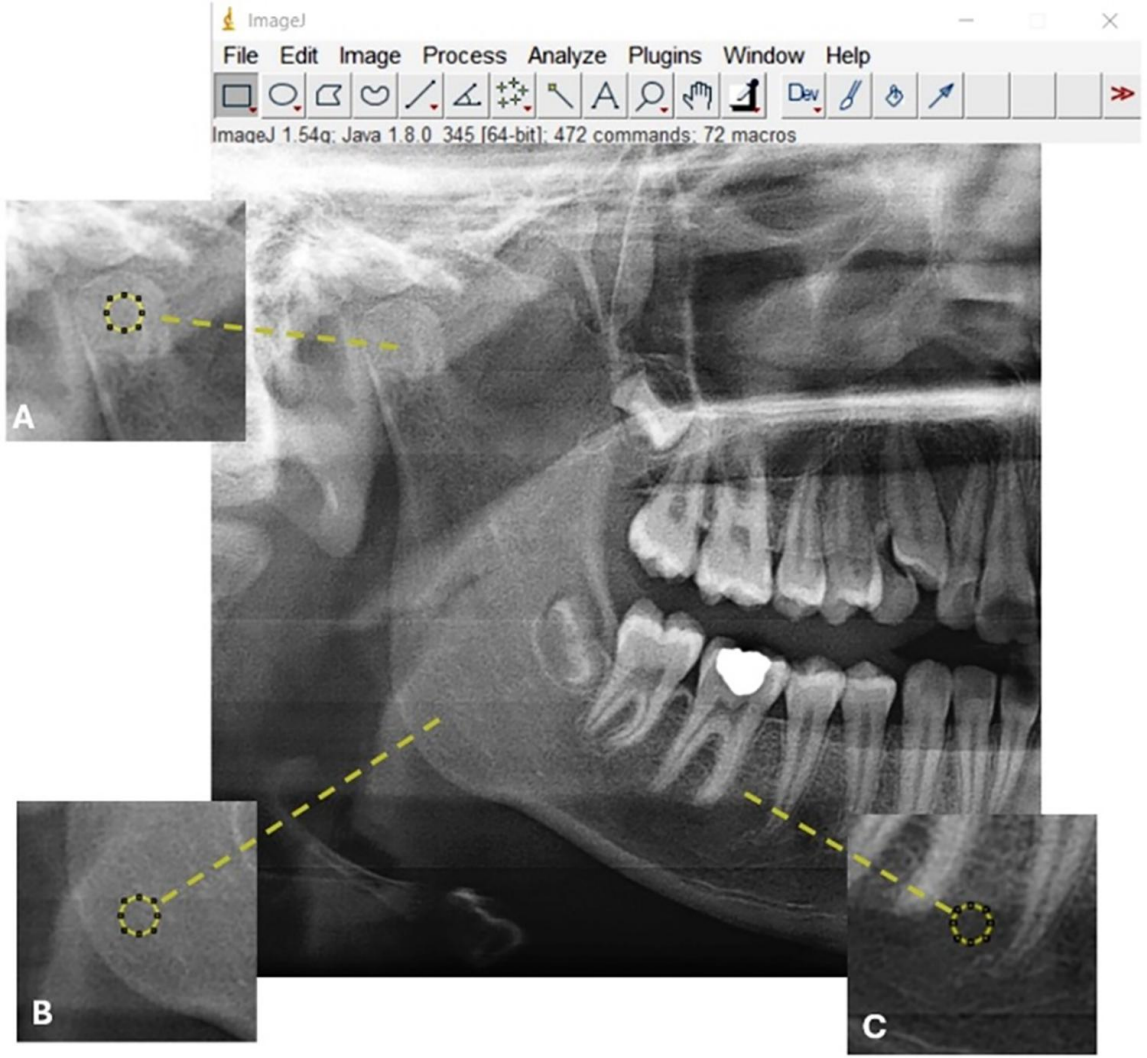
Panoramic radiograph illustrating the three regions of interest (ROIs) selected for fractal dimension analysis. **(A)** ROI-1: 30 × 30 pixel area at the geometric center of the mandibular condyle. **(B)** ROI-2: 30 × 30 pixel area at the geometric center of the mandibular angle. **(C)** ROI-3: 30 × 30 pixel area in the molar region superior to the mental foramen. All ROIs were positioned on the right side of the mandible, excluding teeth, cortical bone, and the mental foramen.

Digitally recorded panoramic images were transferred to ImageJ 1.8v software (National Institutes of Health, Bethesda, MD, USA). Panoramic radiographs used within the scope of the study were obtained using the same brand and model digital panoramic device (RAYSCAN Alfa-P). For FD analysis, three anatomical locations were selected in each image, and a 30 × 30 pixel region of interest (ROI) was defined for each area (Figure 1). To ensure standardization, the right side of the mandible was used in all cases (17), and ROIs were positioned to exclude teeth, cortical bone, and the mental foramen.

These points were:

1. A 30 × 30 pixel area at the geometric center of the condyle (Figure 1A)
2. A 30 × 30 pixel area at the geometric center of the mandibular angle (Figure 1B)
3. A 30 × 30 pixel area in the molar region superior to the mental foramen (Figure 1C)

**Figure 2:**
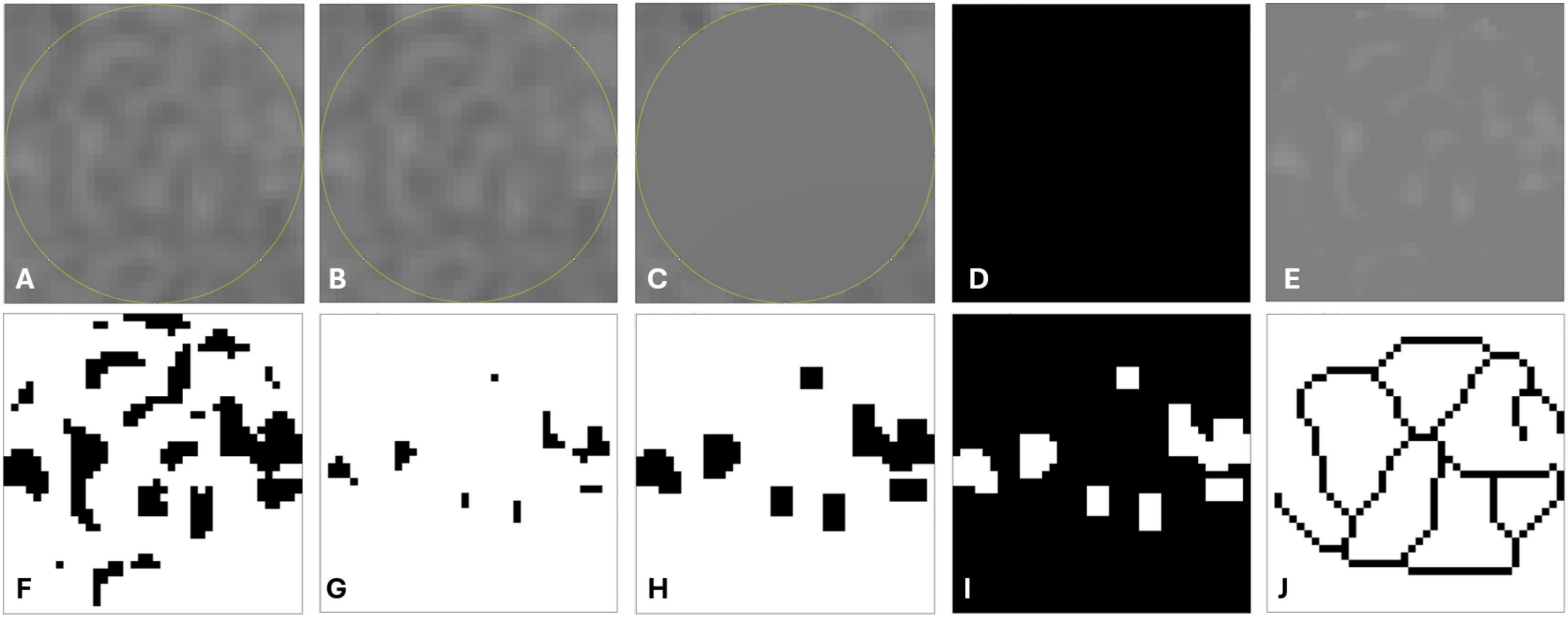
Sequential image processing steps applied for fractal dimension analysis using ImageJ 1.8v software. **(A)** Cropped grayscale image of the region of interest. **(B)** Duplicated image. **(C)** Gaussian blur filter applied (sigma = 35 pixels) to minimize brightness variations caused by overlapping soft tissues and differences in bone thickness. **(D)** Subtracted image to enhance edge visibility. **(E)** Image with added intensity (128) for normalization. **(F)** Binarized (black-and-white) image. **(G)** Eroded image for noise reduction. **(H)** Dilated image to enhance trabecular structures. **(I)** Inverted binary image for improved outline visibility. **(J)** Skeletonized image for structural simplification prior to box-counting analysis.

**Figure 3:**
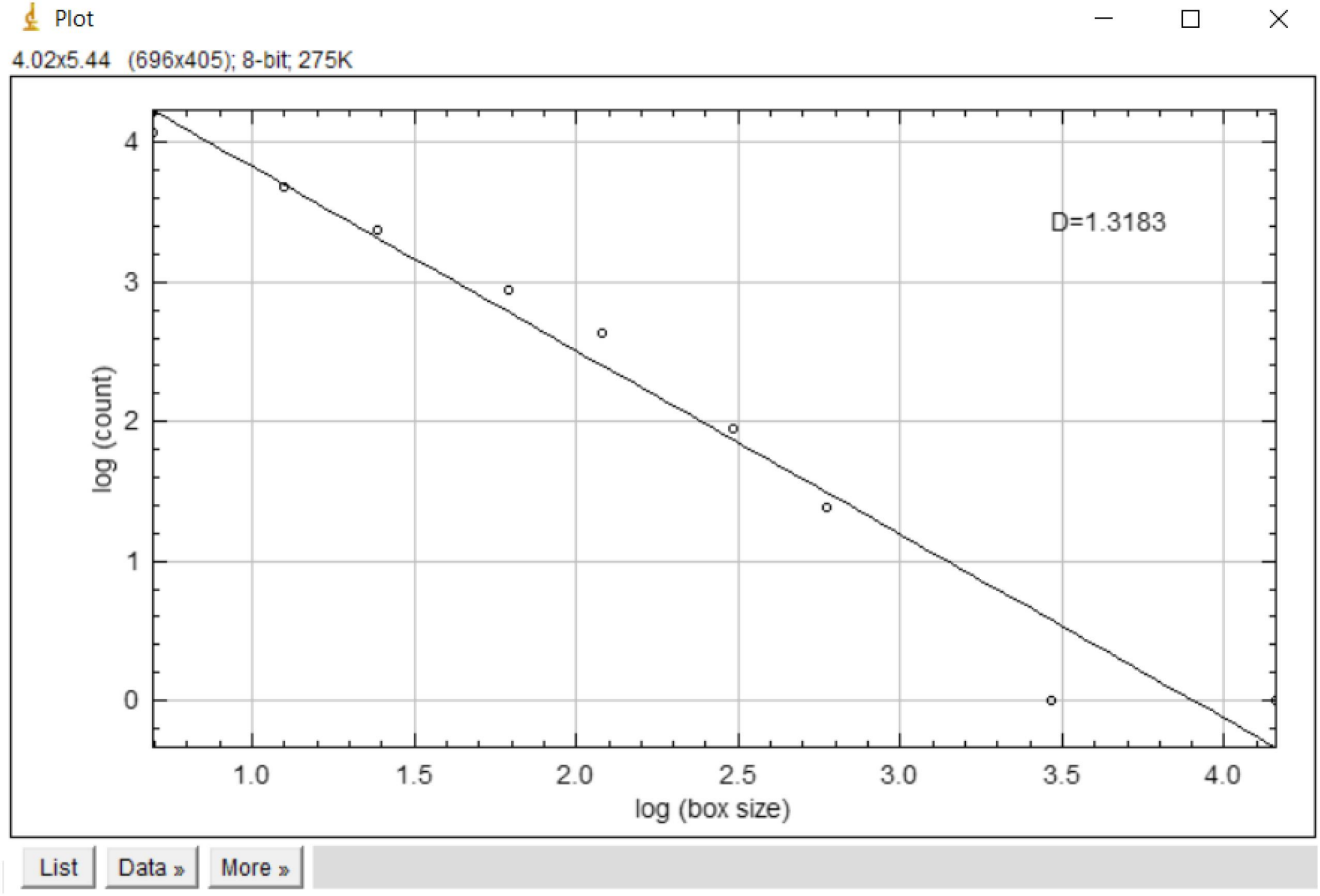
Representative logarithmic graph obtained from the box-counting method, in which the number of boxes containing trabecular structures is plotted against box size. The slope of the best-fit line corresponds to the fractal dimension (FD) value “D”.

FD analysis was performed using the box-counting method following the image processing protocol proposed by White and Rudolph (18) (Figure 2). Each image is divided into squares of 2, 3, 4, 6, 8, 12, 16, 32 and 64 pixels. For each box size, the number of boxes containing trabecular structures and the total number of boxes are computed. A logarithmic graph is plotted based on these values, with the slope of the best-fit line representing the FD value (Figure 3). This value is expressed as the FD value “D”, calculated from the slope of the line that best fits the points obtained from the logarithmic scale graph of the values.

The FD values of trabecular bone calculated using the box-counting method are expected to range between 1 and 2. The FD value of alveolar bone typically falls within this range, indicating that the bone exhibits fractal geometry. Values closer to 2 represent a more complex bone microarchitecture, while values approaching 1 indicate simpler, more porous structures (19). In this context, when FD values obtained from the study are compared between the research and control groups, the presence of a statistically significant difference may indicate substantial variations in trabecular complexity and bone density. The use of FD analysis in assessing bone microarchitecture provides an objective method for detecting potential structural differences between groups.

All fractal dimension measurements were performed by a single examiner who was blinded to the group allocation of the participants at the time of analysis.

### 2.8 Statistical analyses

Statistical analyses were performed using IBM SPSS Statistics 27. The normality of continuous variables was assessed using the Shapiro–Wilk test. Fractal dimension (FD) values in the condyle, angle, and molar regions were normally distributed, whereas caries experience (dmft + DMFT) and CPITN index values were not normally distributed. Independent samples *t*-test was used for normally distributed data, while the Mann–Whitney *U* test was applied for non-normally distributed data. Categorical variables were analyzed using the chi-square test. The Kruskal–Wallis test was employed to determine differences among medication combination groups. The relationship between medication duration and oral findings, dmft + DMFT, and CPITN index was evaluated using Spearman correlation analysis. A *p*-value of less than 0.05 was considered statistically significant for all analyses. The statistical power of the study was assessed based on a 95% confidence level (*α* = 0.05) and 80% power (1−*β* = 0.80).

## 3 Results

### 3.1 Demographic and clinical characteristics

A total of 34 children with IBD (10 with CD and 24 with UC) and 34 age- and gender-matched healthy controls were included in the study. The demographic characteristics of both groups showed no statistically significant differences between the IBD and control groups with respect to age (*p* = 0.936) or gender distribution (*p* = 1.000), confirming that the groups were well-matched (Table 1).

**Table 1:** Demographic characteristics of the study groups.

Characteristic	IBD group (*n* = 34)	Control group (*n* = 34)	Test statistic	*p*
Age—median (Min–Max)	15.3 (7.0–19.8)	15.5 (7.0–19.0)	*U* = 571	0.936
Gender—female, *n* (%)	20 (58.8%)	21 (61.8%)	–	1.000
Gender—male, *n* (%)	14 (41.2%)	13 (38.2%)	–	

IBD, inflammatory bowel disease; Min–Max, minimum–maximum; *U*, Mann–Whitney *U* test.

The most commonly used drug groups were 5-ASA (74%) and immunomodulators (IMM, 68%), and the most frequent combination was 5-ASA with IMM (47%), reflecting standard IBD treatment practice (Table 2). As patients could receive more than one drug group simultaneously, percentages exceed 100% in total.

**Table 2:** Drug combination profiles of the study group.

Drug group co-usage matrix (*n* = 34)						
Drug group	5-ASA	IMM	COR	TNF	IL-23	JAK
5-ASA	*n* = 25 (74%)	*n* = 16 (47%)	*n* = 3 (9%)	*n* = 3 (9%)	*n* = 1 (3%)	*n* = 1 (3%)
IMM	*n* = 16 (47%)	*n* = 23 (68%)	*n* = 3 (9%)	*n* = 1 (3%)	–	*n* = 2 (6%)
COR	*n* = 3 (9%)	*n* = 3 (9%)	*n* = 4 (12%)	–	–	*n* = 1 (3%)
TNF	*n* = 3 (9%)	*n* = 1 (3%)	–	*n* = 4 (12%)	–	–
IL-23	*n* = 1 (3%)	–	–	–	*n* = 2 (6%)	–
JAK	*n* = 1 (3%)	*n* = 2 (6%)	*n* = 1 (3%)	–	–	*n* = 2 (6%)

Each cell shows the number (*n*) and percentage (%) of patients using two drug groups simultaneously. Diagonal cells (shaded): total number of patients using the relevant drug group. Other cells: patients using both groups concurrently. 5-ASA, 5-aminosalicylic acid; IMM, immunomodulator (azathioprine); COR, corticosteroid; TNF, anti-TNF biologic (infliximab, adalimumab); IL-23, anti-IL-23 biologic; JAK, JAK inhibitor.

### 3.2 Caries experience and periodontal status

No statistically significant difference was found between the IBD and control groups in terms of either total caries experience (*p* = 0.173) or CPITN index (*p* = 0.219) (Table 3).

**Table 3:** Comparison of total caries experience (dmft + DMFT) and CPITN index between study groups.

Variable	IBD group (*n* = 34) median (Min–Max)	Control group (*n* = 34) median (Min–Max)	Test statistic	*p*
dmft + DMFT	4.5 (0–15)	3.5 (0–14)	689.0	0.173
CPITN index	1.0 (0–2)	1.0 (0–2)	669.5	0.219

IBD, inflammatory bowel disease; dmft, decayed, missing, and filled primary teeth; DMFT, decayed, missing, and filled permanent teeth; CPITN, community periodontal index of treatment needs; Min–Max, minimum–maximum; *U*, Mann–Whitney *U*.

### 3.3 Mandibular trabecular bone fractal dimension

Statistically significant differences were found between the IBD and control groups in the condyle (*p* = 0.047; Cohen's *d* = −0.49, 95% CI −0.98 to −0.01) and molar (*p* < 0.001; Cohen's *d* = −1.13, 95% CI −1.64 to −0.62) regions, with the IBD group consistently showing lower FD values. No significant difference was observed in the angle region (*p* = 0.272; Cohen's *d* = −0.33, 95% CI −0.81 to 0.15) (Table 4).

**Table 4:** Comparison of mandibular trabecular bone fractal analysis (FD) values between study groups.

Region (ROI)	IBD group (*n* = 34)		Control group (*n* = 34)		Test statistic	*p*
	Min–Max	Mean ± SD (median)	Min–Max	Mean ± SD (median)		
ROI 1-condyle	1.11–1.40	1.32 ± 0.07 (1.34)	1.24–1.41	1.35 ± 0.05 (1.36)	−2.022	0.047*
ROI 2-angle	1.20–1.40	1.30 ± 0.05 (1.31)	1.15–1.41	1.32 ± 0.07 (1.34)	−1.107	0.272
ROI 3-molar	1.17–1.38	1.31 ± 0.04 (1.32)	1.28–1.40	1.35 ± 0.03 (1.35)	−4.737	<0.001*

IBD, inflammatory bowel disease; ROI, region of interest; FD, fractal dimension; SD, standard deviation; Min–Max, minimum–maximum.

* *p* < 0.05, independent samples *t*-test.

Of the 34 children with IBD, 10 were diagnosed with CD and 24 with UC. A statistically significant difference was observed only in the condyle region (*p* = 0.046; Cohen's *d* = −0.89, 95% CI −1.66 to −0.12), where children with CD exhibited lower FD values compared to those with UC. No significant differences were detected in the angle or molar regions (*p* > 0.05) (Table 5).

**Table 5:** Comparison of fractal analysis values by disease type (Crohn's disease/ulcerative colitis).

Region (ROI)	Crohn (*n* = 10)		Ulcerative colitis (*n* = 24)		Test statistic	*p*
	Min–Max	Mean ± SD (median)	Min–Max	Mean ± SD (median)		
ROI 1- condyle	1.11–1.40	1.28 ± 0.11 (1.33)	1.24–1.39	1.34 ± 0.04 (1.34)	−2.081	0.046*
ROI 2-angle	1.20–1.39	1.30 ± 0.05 (1.32)	1.21–1.40	1.30 ± 0.05 (1.29)	−0.150	0.882
ROI 3-molar	1.20–1.36	1.31 ± 0.05 (1.33)	1.17–1.38	1.31 ± 0.04 (1.32)	−0.127	0.899

ROI, region of interest; SD, standard deviation; Min–Max, minimum–maximum.

* *p* < 0.05, independent samples *t*-test.

No statistically significant difference was detected among the groups in any of the three ROIs (ROI 1: *p* = 0.395; ROI 2: *p* = 0.637; ROI 3: *p* = 0.179), suggesting that the type of medication combination used did not significantly influence mandibular trabecular bone microarchitecture in the study group (Table 6).

**Table 6:** Comparison of mandibular trabecular bone fractal dimension (FD) values by medication combination groups.

Drug combination	*n*	ROI 1—condyle	ROI 2—angle	ROI 3—molar	*H*	*p*
		*n* ≥ 3: Mean ± SD (median) *n* = 2: mean (min–max) *n* = 1: value	*n* ≥ 3: Mean ± SD (median) *n* = 2: mean (min–max) *n* = 1: value	*n* ≥ 3: Mean ± SD (median) *n* = 2: mean (min–max) *n* = 1: value		
5-ASA + IMM	13	1.34 ± 0.05 (1.36)	1.29 ± 0.04 (1.32)	1.32 ± 0.03 (1.33)	ROI 1: 12.655 ROI 2: 9.756 ROI 3: 16.274	0.395 0.637 0.179
IMM	5	1.32 ± 0.11 (1.37)	1.30 ± 0.07 (1.30)	1.30 ± 0.07 (1.33)		
5-ASA	5	1.35 ± 0.01 (1.34)	1.33 ± 0.06 (1.37)	1.29 ± 0.01 (1.30)		
5-ASA + TNF	2	1.26 (1.24–1.28)	1.34 (1.28–1.40)	1.35 (1.32–1.38)		
5-ASA + IMM + TNF	1	1.27	1.32	1.30		
5-ASA + IMM + COR + JAK	1	1.34	1.29	1.29		
IMM + COR	1	1.16	1.36	1.28		
IMM + JAK	1	1.30	1.27	1.20		
IL-23	1	1.11	1.25	1.25		
5-ASA + IMM + COR	1	1.32	1.32	1.28		
TNF	1	1.36	1.39	1.35		
5-ASA + COR	1	1.30	1.29	1.36		
5-ASA + IL-23	1	1.37	1.28	1.30		

*H*, Kruskal–Wallis; ROI, region of interest; IMM, immunomodulator; COR, corticosteroid; TNF, anti-TNF biologic; JAK, JAK inhibitor; 5-ASA, 5-aminosalicylic acid, Min–Max, minimum–maximum.

### 3.4 Intraoral lesions and medication duration

Intraoral lesions were more prevalent in children with CD (90.0%) compared to those with UC (37.5%). Among lesion types, cobblestone appearance of the buccal mucosa was observed exclusively in children with CD, while aphthous ulcers were the most common finding in both groups (Table 7).

**Table 7:** Distribution of intraoral lesions by disease type in the study group.

Finding	Crohn (*n* = 10)	Ulcerative colitis (*n* = 24)
	*n* (%)	*n* (%)
Intraoral lesion		
Present	9 (90.0%)	9 (37.5%)
Absent	1 (10.0%)	15 (62.5%)
Lesion type		
Aphthous ulcer	7 (77.8%)	8 (88.9%)
Cobblestone appearance of buccal mucosa	2 (22.2%)	0 (0.0%)
Cheilitis	1 (11.1%)	2 (22.2%)
Candida	0 (0.0%)	1 (11.1%)

As more than one lesion type may be present in a single patient, the sum of lesion type percentages may exceed 100%.

No statistically significant correlation was found between medication duration and any of the intraoral findings across all drug groups evaluated (*p* > 0.05). IL-23 (*n* = 2) and JAK (*n* = 2) groups were excluded from the analysis due to insufficient sample size (Table 8).

**Table 8:** Correlation between medication duration and intraoral findings.

Oral finding	5-ASA (*n* = 24)		IMM (*n* = 23)		COR (*n* = 4)		TNF (*n* = 4)	
	*r*	*p*	*r*	*p*	*r*	*p*	*r*	*p*
Lesion presence (yes/no)	0.191	0.373	−0.135	0.540	0.894	0.106	0.000	1.000
dmft + DMFT	0.292	0.166	0.182	0.407	0.000	1.000	0.316	0.684
CPITN index	−0.256	0.228	−0.032	0.886	−0.105	0.895	0.447	0.553

Spearman correlation test. *r*, correlation coefficient; IMM, immunomodulator; COR, corticosteroid; TNF, anti-TNF biologic; 5-ASA, 5-aminosalicylic acid; dmft, decayed, missing, and filled primary teeth; DMFT, decayed, missing, and filled permanent teeth; CPITN, community periodontal index of treatment needs.

## 4 Discussion

This study is the first to evaluate mandibular trabecular bone microarchitecture using fractal dimension (FD) analysis in children with IBD, and it yielded several clinically relevant findings. Most notably, children with IBD exhibited significantly lower FD values in the condyle (*p* = 0.047) and molar (*p* < 0.001) regions than healthy controls, indicating a measurable deterioration in mandibular trabecular bone complexity. This impairment was more pronounced in CD than in UC, with children with CD showing significantly lower condylar FD values (*p* = 0.046). In parallel, oral mucosal involvement was markedly more common in CD, where intraoral lesions reached a prevalence of 90.0% and aphthous ulcers represented the most frequent finding. By contrast, caries experience and periodontal status did not differ significantly between the IBD and control groups (*p* = 0.173 and *p* = 0.219, respectively), and medication duration showed no significant association with oral findings (*p* > 0.05). These findings are discussed below in the context of the existing literature.

Studies evaluating children with IBD have largely focused on intraoral findings (20, 21) and dental caries and periodontal treatment needs (6, 7). Koutsochristou et al. (6) reported that children and adolescents with IBD had significantly higher dmft/DMFT indices, increased gingival inflammation, and greater periodontal treatment needs compared to healthy controls, while no significant difference was found between groups regarding plaque accumulation. Haznedaroğlu and Polat (7) compared the prevalence of dental caries, dental erosion, and periodontal disease in children with IBD against healthy controls, finding a higher frequency of oral mucosal findings and periodontal disease in the IBD group, whereas the DMFT index did not differ significantly between groups. In the present study, no statistically significant difference was observed between the IBD and control groups in terms of dmft + DMFT or CPITN index (*p* = 0.173 and *p* = 0.219, respectively). Our findings are consistent with those of Haznedaroğlu and Polat (7), who similarly reported no significant difference in DMFT between groups, but contrast with the results of Koutsochristou et al. (6), who observed significantly higher caries experience and periodontal treatment needs in patients with IBD. This discrepancy may be partly attributable to the relatively limited sample size of the present study, as other investigations in this field were conducted with larger patient cohorts. Furthermore, factors such as dietary habits, socioeconomic status, and oral hygiene behaviors were not assessed in the present study, which may have limited the detection of intergroup differences.

Oral manifestations of IBD have long been recognized as clinically significant extraintestinal findings. In a study conducted by Klichowska-Palonka et al. (20), intraoral findings in children diagnosed with IBD were compared against healthy children, with aphthous stomatitis and angular cheilitis identified as the most frequently observed oral lesions in the IBD group. In a prospective study by Harty et al. (21), oral findings at the time of diagnosis were evaluated in children with CD, with disease-specific oral involvement identified in more than 40% of patients. The most commonly reported lesions were mucogingivitis, mucosal tags, deep ulcerations, and cobblestone mucosa, highlighting the oral cavity as a potentially important site for diagnostic evaluation. Consistent with these findings, the majority of children with IBD in the present study had at least one intraoral lesion, with aphthous ulcers being the most common finding. No intraoral lesions were observed in any of the control group patients. Regarding disease subtype, the prevalence of intraoral lesions was notably higher in the CD group compared to the UC group (90.0% vs. 37.5%), and cobblestone appearance of the buccal mucosa was observed exclusively in patients with CD. These findings suggest that CD may be associated with a higher frequency and greater variety of oral manifestations compared to UC, and support the importance of thorough oral cavity evaluation as part of the clinical follow-up of patients with IBD.

Fractal dimension analysis is emerging as an increasingly recognized method for the objective evaluation of changes in mandibular trabecular bone microarchitecture using panoramic radiographs. Fractal analysis has been reported as an effective tool for the quantitative assessment of qualitative changes in trabecular bone structure in various systemic diseases, including hyperparathyroidism, osteoporosis, and rheumatoid arthritis (22–24). Türkmenoğlu et al. (24) evaluated condylar trabecular FD in 100 patients with rheumatoid arthritis, classified as normal, osteopenic, or osteoporotic by *T*-score, along with age- and gender-matched healthy controls. FD analysis distinguished healthy condyles from those affected by rheumatoid arthritis even when bone mineral density was preserved. Sevimay et al. (25) assessed mandibular trabecular bone microarchitecture using FD analysis in 73 patients categorized into three groups—23 with osteoporosis, 25 receiving antiresorptive therapy for oncologic conditions, and 25 systemically healthy controls—and reported that FD values in the molar region were significantly lower in osteoporosis patients compared to healthy controls. A similar pattern was observed in the present study, with FD values in the molar and condyle regions being significantly lower in children with IBD compared to healthy controls. This finding supports the adverse impact of alterations in bone metabolism on mandibular trabecular structure and suggests that FD analysis may serve as a sensitive and non-invasive diagnostic tool in this field.

Reduced bone mineral density (BMD) has been frequently reported in children with IBD (26). A study conducted in Korea among newly diagnosed children with IBD revealed that a substantial proportion of patients already exhibited low BMD prior to the initiation of treatment, emphasizing that reduced BMD may emerge independently of disease-specific factors at an early stage (27). Although prolonged glucocorticosteroid use has been identified as a major clinical risk factor for low BMD, Paganelli et al. (28) reported that neither the cumulative dose nor the duration of corticosteroid therapy correlated significantly with Bone Mineral Apparent Density (BMAD), concluding that inflammatory activity is the primary determinant of bone loss, independent of medication exposure. The absence of a significant effect of medication combination or duration on FD values in the present study diverges from the findings of Schmidt et al. (26) regarding azathioprine as a risk factor, yet is consistent with those of Paganelli et al. (28). This discrepancy may be partly explained by the small number of patients receiving corticosteroids in our cohort (*n* = 4). The declining use of corticosteroids in pediatric IBD, together with the growing role of biologics and immunomodulators, may also have altered the pattern of drug-related bone loss.

This study is among the first to evaluate the effects of medications used in children with IBD on both intraoral findings and mandibular bone microarchitecture. Although Haznedaroğlu and Polat (7) emphasized that oral manifestations in children with IBD may be related to medication-induced reactions or nutritional deficiencies, they did not directly assess the association between medication duration and oral findings. In this regard, the present study represents a novel contribution by simultaneously evaluating the impact of medication type, combination, and duration on intraoral findings and mandibular trabecular bone microarchitecture in this patient population. No statistically significant correlation was found between medication duration and intraoral findings across all drug groups evaluated (*p* > 0.05). Nevertheless, the negative correlation coefficients observed between 5-ASA duration and CPITN index (*r* = −0.256), and between immunomodulator duration and lesion presence (*r* = −0.135), suggest a trend toward reduced periodontal treatment needs and lower lesion prevalence with longer medication use; however, these associations did not reach statistical significance.

The study has several limitations. First, its cross-sectional design and the inclusion of patients who had already been diagnosed and started on treatment introduced considerable variability in disease duration and medication exposure, precluding the establishment of causal relationships and making it difficult to distinguish disease-related from treatment-related changes in FD values. Second, the relatively small overall sample (*n* = 68) and the low numbers in certain medication subgroups—including IL-23 (*n* = 2) and JAK (*n* = 2) inhibitors, which could not be analyzed—reduced statistical power. Another limitation is that disease activity was not systematically assessed, and potential confounders affecting bone metabolism and oral health, such as vitamin D status, body mass index, pubertal stage, oral hygiene habits, nutritional status, and socioeconomic level, were not evaluated.

## 5 Conclusion

Mandibular trabecular bone microarchitecture, assessed by fractal dimension analysis, was significantly impaired in children with IBD compared to healthy controls, particularly in the condylar and molar regions. Children with CD exhibited a higher prevalence of intraoral lesions and lower condylar FD values than those with UC, whereas caries experience, periodontal status, and oral or radiographic findings were not significantly influenced by medication type, combination, or duration. The long-term effects of biologic and immunomodulatory therapies on mandibular bone microarchitecture warrant further investigation. Applied to readily available panoramic radiographs, fractal dimension analysis offers a practical, low-cost, non-invasive first-line screening tool for detecting mandibular trabecular bone changes in children with IBD, helping to avoid unnecessary exposure to higher-radiation modalities such as CBCT; combining it with DXA may further refine assessment in selected cases.

## Data Availability

The datasets presented in this article are not readily available because of ethical restrictions and patient confidentiality. Requests to access the datasets should be directed to the corresponding author.
